# Membrane estrogen receptor-α-mediated nongenomic actions of phytoestrogens in GH_3_/B_6_/F_10 _pituitary tumor cells

**DOI:** 10.1186/1750-2187-4-2

**Published:** 2009-04-28

**Authors:** Yow-Jiun Jeng, Mikhail Y Kochukov, Cheryl S Watson

**Affiliations:** 1 Department of Biochemistry and Molecular Biology, University of Texas Medical Branch, Galveston, Texas, USA

## Abstract

**Background:**

Estradiol (E_2_) mediates various intracellular signaling cascades from the plasma membrane via several estrogen receptors (ERs). The pituitary is an estrogen-responsive tissue, and we have previously reported that E_2 _can activate mitogen-activated protein kinases (MAPKs) such as ERK1/2 and JNK1/2/3 in the membrane ERα (mERα)-enriched GH_3_/B_6_/F_10 _rat pituitary tumor cell line. Phytoestrogens are compounds found in plants and foods such as soybeans, alfalfa sprouts, and red grapes. They are structurally similar to E_2 _and share a similar mechanism of action through their binding to ERs. Phytoestrogens bind to nuclear ERs with a much lower affinity and therefore are less potent in mediating genomic responses. However, little is known about their ability to act via mERs to mediate nongenomic effects.

**Methods:**

To investigate the activation of different nongenomic pathways, and determine the involvement of mERα, we measured prolactin (PRL) release by radio-immunoassay, MAPK activations (ERK1/2 and JNK1/2/3) via a quantitative plate immunoassay, and intracellular [Ca^2+^] by Fura-2 fluorescence imaging in cells treated with E_2 _or four different phytoestrogens (coumestrol, daidzein, genistein, and *trans*-resveratrol).

**Results:**

Coumesterol and daidzein increased PRL release similar to E_2 _in GH_3_/B_6_/F_10 _cells, while genistein and *trans*-resveratrol had no effect. All of these compounds except genistein activated ERK1/2 signaling at 1–10 picomolar concentrations; JNK 1/2/3 was activated by all compounds at a 100 nanomolar concentration. All compounds also caused rapid Ca^2+ ^uptake, though in unique dose-dependent Ca^2+ ^response patterns for several aspects of this response. A subclone of GH_3 _cells expressing low levels of mERα (GH_3_/B_6_/D_9_) did not respond to any phytoestrogen treatments for any of these responses, suggesting that these nongenomic effects were mediated via mERα.

**Conclusion:**

Phytoestrogens were much more potent in mediating these nongenomic responses (activation of MAPKs, PRL release, and increased intracellular [Ca^2+^]) via mERα than was previously reported for genomic responses. The unique non-monotonic dose responses and variant signaling patterns caused by E_2 _and all tested phytoestrogens suggest that complex and multiple signaling pathways or binding partners could be involved. By activating these different nongenomic signaling pathways, phytoestrogens could have significant physiological consequences for pituitary cell functions.

## Background

Binding of 17β-estradiol (E_2_) to estrogen receptors (ERs) elicits important changes in growth, differentiation, maturation, and other functions of target tissues [1-4]. In the pituitary, E_2 _and its pharmaceutical mimic diethylstilbesterol (DES) have many effects, including proliferation of several pituitary cell types, formation of new blood vessels [5], regulation of most pituitary hormones [6], and tumorigenesis [7]. For some of these effects, the ERs α and β function as ligand-dependent transcription factors controlling expression of responsive genes upon activation. However, there are now numerous studies showing that E_2 _can rapidly influence cellular physiology in pituitary and many other cell types via the activation of a diverse array of intracellular non-nuclear signaling mechanisms [4,8,9]. For example, E_2 _can rapidly increase intracellular Ca^2+ ^or activate extracellular regulated kinases (ERK1/2) [10,11]. Although the complete mechanistic details of these nongenomic actions are not fully understood, it is known that some of the rapid E_2 _effects are initiated by binding of E_2 _at membrane-associated ERs that are closely related to the "classical" intracellular ERs [12-14].

Phytoestogens are a group of lipophilic plant compounds that are structurally similar to E_2 _and have been shown to bind to ERs (α and β) and the alternative ER, GPR30 [15-17]. Both genomic and nongenomic mechanisms have been proposed to mediate phytoestrogenic effects. Phytoestrogens can bind to ERs and induce expression of specific estrogen-responsive gene products in conjunction with regulating cellular processes such as growth and differentiation [18,19]. We found previously that coumesterol, a phytoestrogen with relatively high estrogenic potency for nuclear actions [20], can also initiate signaling from the membrane such as activation of ERK1/2, PRL release, and changes in Ca^2+ ^fluxes [21,22] in rat pituitary cells. Rapid activations by both E_2 _and phytoestrogens via mERs have also been reported in other tissues, such as brain [23]. In the present study, we extend these observations to several other phytoestrogens, in comparison to E_2 _and coumesterol. Coumesterol can disrupt reproduction in livestock [24], where its major dietary sources are legumes, clover, and alfalfa sprouts. Serum concentrations of coumesterol can be as high as 0.01 μM in humans [25]. The popular food supplements isoflavones are soybean components, and are represented by daidzein and genistein in our studies. Differences between Western and Asian diets may underlie the population variations in the incidence of estrogen-influenced cancers and other diseases in a variety of tissues [26]. Consumption of Asian-style diets enriched in these soybean products can lead to plasma concentrations of isoflavonoids of 0.1 to 10 μM [20,25]. Resveratrol, a stilbene which is found in high quantities in foods such as red grapes (and wine) and peanuts has two isomers (*cis *and *trans*), but only the *trans *form has been reported to be estrogenic [27]. There is growing evidence that resveratrol may prevent or delay the onset of cancer, heart disease, diabetes, pathological inflammations and viral infections [28]. The human serum concentrations of resveratrol and its metabolites are variable, but can peak at close to 2 μM [29].

Nongenomic effects caused by phytoestrogens could play a key role in a variety of biological functions by altering membrane-initiated signaling patterns. E_2_-mediated activation of kinases in the MAPK family (including ERK1/2 and JNK1/2/3) can change the complex partnering of ERs with other kinases, such as Src and Ras [30]. In addition, downstream cytoplasmic or transcriptional events have been attributed to activation of MAPKs [31]. E_2 _can also rapidly regulate intracellular Ca^2+ ^levels [11,32], important for triggering release of secretory granules, maintaining homeostatic functions and regulating other estrogen-induced kinase changes [33]. A rapid, stimulatory effect of E_2 _on prolactin (PRL) secretion could affect milk production, osmoregulation, and maternal behaviors [6]. In the current study we used a wide range of concentrations of different phytoestrogens to elicit rapid kinase activations, PRL release, and elevation of intracellular [Ca^2+^] in the mERα-enriched rat pituitary cell line GH_3_/B_6_/F_10 _[34]. We also examined the involvement of mERα by comparing these nongenomic responses in the mERα-depleted cell line GH_3_/B_6_/D_9_. Our elucidation of the distinct abilities of different phytoestrogens to activate these nongenomic signaling pathways in different dose-response patterns could predict the physiological consequences of these compounds as dietary components or hormone replacement supplements.

## Methods

### Materials and Cells

We purchased phenol red-free Dulbecco Modified Eagle Medium (DMEM) from Mediatech (Herndon, VA); horse serum from Gibco BRL (Grand Island, NY); defined supplemented calf sera and fetal bovine sera from Hyclone (Logan, UT). Paraformaldehyde and glutaraldehyde were purchased from Fisher Scientific (Pittsburgh, PA). We purchased Fura-2 AM from Molecular Probes (Eugene, OR). Antibodies (Abs) used in the ERK and JNK phosphorylation studies (individually described below) were purchased from Cell Signaling (Danvers, MA). All other materials were purchased from Sigma (St. Louis, MO).

GH_3_/B_6_/F_10 _(mERα-enriched) and GH_3_/B_6_/D_9 _(mERα-poor) cell sublines [34] were routinely cultured in DMEM containing 12.5% horse serum, 2.5% defined supplemented calf serum, and 1.5% fetal calf serum. They were then switched to various hormone-free media prior to our experiments, as described below. Cells were used between passages 10 and 20.

### PRL release measured via RIA

Cells (0.5–0.7 × 10^6^) were plated in poly-D-lysine-coated six-well plates. After serum deprivation in ITS defined medium [5 μg/mL insulin, 5 μg/mL transferring, 5 ng/mL sodium selenite, and 0.1% bovine serum albumin (BSA)] for 48 h, this medium was removed and DMEM/0.1% BSA was added with or without the appropriate estrogen or vehicle control (ethanol). The cells were incubated for 2 min and centrifuged at 4°C (350 × *g *for 5 min), then the supernatant was collected and stored at -20°C. The PRL concentrations were determined using components of the rat PRL RIA kit from the National Institute of Diabetes and Digestive and Kidney Disease and the National Hormone and Pituitary Program (Baltimore, MD). Briefly, RIA buffer [80% phosphate-buffered saline (PBS), 20% DMEM, 2% normal rabbit serum], 100 μL cold standard (rat PRL-RP-3) or unknown sample, rPRL-s-9 antiserum (final dilution of 1:437,500 in RIA buffer), and [^125^I]-rat-PRL (PerkinElmer, Wellesley, MA, USA; using 15,000 counts per tube diluted in RIA buffer) were combined and incubated with shaking overnight at 4°C. Anti-rabbit IgG (R-0881; Sigma) was added to a final dilution of 1:9, and the samples were incubated with shaking at RT for 2 h. One mL of polyethylene glycol solution [1.2 M polyethylene glycol (P-6667; Sigma), 50 mM Tris, pH 8.6] was then added, the samples were incubated with shaking at RT for 15 min, then centrifuged at 4,000 × *g *for 10 min at 4°C. The supernatant was decanted, and the pellet counted in a Wizard 1470 Gamma Counter (PerkinElmer, Boston, MA). The PRL concentration was then calculated and normalized to the crystal violet (CV) values estimating the number of cells in each well [35]. To perform the CV assay cells were stained with 0.1% CV solution and incubated at room temperature for 30 min, then washed with PBS and dried. The dye was then extracted with 10% acetic acid solution and read at A_590 _nm. These measurements (n = 6) were done in 3 different experiments on different days.

### ERK and JNK assays

We originally developed this assay to assess activated ERK 1/2 levels in fixed GH_3_/B_6 _cell subclones [10], and subsequently adapted it to equivalent assays for JNK1/2/3. Briefly, cells were plated at a density of 10,000 cells/well in poly-D-lysine-coated 96-well plates. Growth media were replaced on the following day (for 48 h) with DMEM containing 1% charcoal-stripped (4×) serum (to deprive cells of steroids). Cells were then washed with DMEM once before the estrogens (or 0.0001% ethanol vehicle) were added for 5 (ERK) or 15 min (JNK). The cells were then fixed with 2% paraformaldehyde/0.2% picric acid at 4°C for 48 h. After fixation, the cells were permeabilized with PBS containing 2% BSA and 0.1% Triton X-100 for 1 h at RT, then washed 3× with PBS; primary antibody (Ab) against phosphorylated ERKS (Cell Signaling Technology, p-Thr202/Tyr204; 1:400 in PBS/1% BSA) or phosphorylated JNK (Cell Signaling Technology, p-Thr183/Tyr185; 1:500 in PBS/1% BSA) was then added. After overnight incubation at 4°C, the cells were washed 3× with PBS, and the biotin-conjugated secondary Ab (Vector Labs, Burlingame CA, 1:300) in PBS/1% BSA was added for 1 h at RT. The cells were again washed with PBS, incubated with Vectastain ABC-AP solution (Vector Labs) for 1 h at RT, and again washed 3× with PBS, followed by addition of Vectastain alkaline phosphatase substrate plus levamasole (an endogenous phosphatase inhibitor). Plates were incubated in the dark for 30 min at 37°C, and the signal for the phosphatase product *para*-nitrophenol was read at A_405 _in a model 1420 Wallac microplate reader (Perkin Elmer, Waltham, MA). The number of cells in each well was estimated by the CV assay and used to normalize phosphorylated enzyme values in individual wells. The experiments (n = 8) were repeated at least 3 times using different passages of cells on different days.

### Ca^2+ ^imaging

Cells were plated on poly-D-lysine-treated 35/22 mm glass-bottom dishes (Willco Wells, Amsterdam, Netherlands) at a density of 100,000 cells/mm^3^. After 48–72 h, the cells were incubated in serum-free DMEM for 12 h before being loaded with 2.5 μM of the Ca^2+^-sensitive fluorescent dye Fura-2 AM for 1 h at RT. They were then washed 3 times, and incubated at RT for 1–4 h before live Ca^2+ ^imaging experiments. The physiological solution used for Fura-2 AM loading and live-cell imaging contained 150 mM NaCl, 5.5 mM KCl, 1 mM MgCl_2_, 4 mM CaCl_2_, 7 mM glucose, and 10 mM HEPES, pH 7.4.

The cell imaging setup included a Nikon 200E microscope with 40× SuperFluo lens and computer-controlled illumination system (Sutter Instruments, Novato, CA) equipped with a digital monochrome-cooled charge-coupled device Roper Coolsnap HQ camera (Roper Scientific, Tucson, AZ).

Fluorescent emissions at 510 nm were acquired from regions corresponding to a single cell with the MetaFluor software (Universal Imaging, Downington, PA) using dual 340 and 380 nm excitation mode. The average intensity of fluorescence in each region was used to estimate 340:380 ratios (R), reflecting [Ca^2+^].

MetaMorph (Universal Imaging, Downington, PA) and SigmaPlot (Systat Software, San Jose, CA) scientific software were used for conversion and analysis of acquired data. The AutoFit function of PeakFit software (Systat Software, San Jose, CA) with manual adjustments was used to quantitatively characterize Ca^2+ ^oscillation amplitude, volume, and frequency in live cells. The peak threshold was chosen empirically as ΔR ≥0.05. Individual cells were considered responsive to hormone treatments when they demonstrated an increase in Ca^2+ ^oscillation frequency compared to basal, showed at least 0.25 Ca^2+ ^spikes per min as estimated over a 10 min time interval, and with a delay of no more than 10 min from the addition of each estrogen into the bath. Subsequent comparative analysis of cell responses was performed on these defined responsive cells only. The experiments were repeated using different passages of cells on different days. Ca^2+ ^traces of at least 30 cells were examined for each condition.

### Statistics

Data from MAPK assays and intracellular Ca^2+ ^measurements were analyzed by one-way analysis of variance (ANOVA) followed by multiple comparisons vs. control group (Holm-Sidak method for MAPK assay; Dunnett's and Duncan's method for intracellular Ca^2+^). The Sigma Stat 3 program (Systat Software, Inc.) was used for all statistical analysis, and significance was accepted at p < 0.05.

## Results

### PRL release caused by E_2 _and different phytoestrogens (Figure 1)

**Figure 1:**
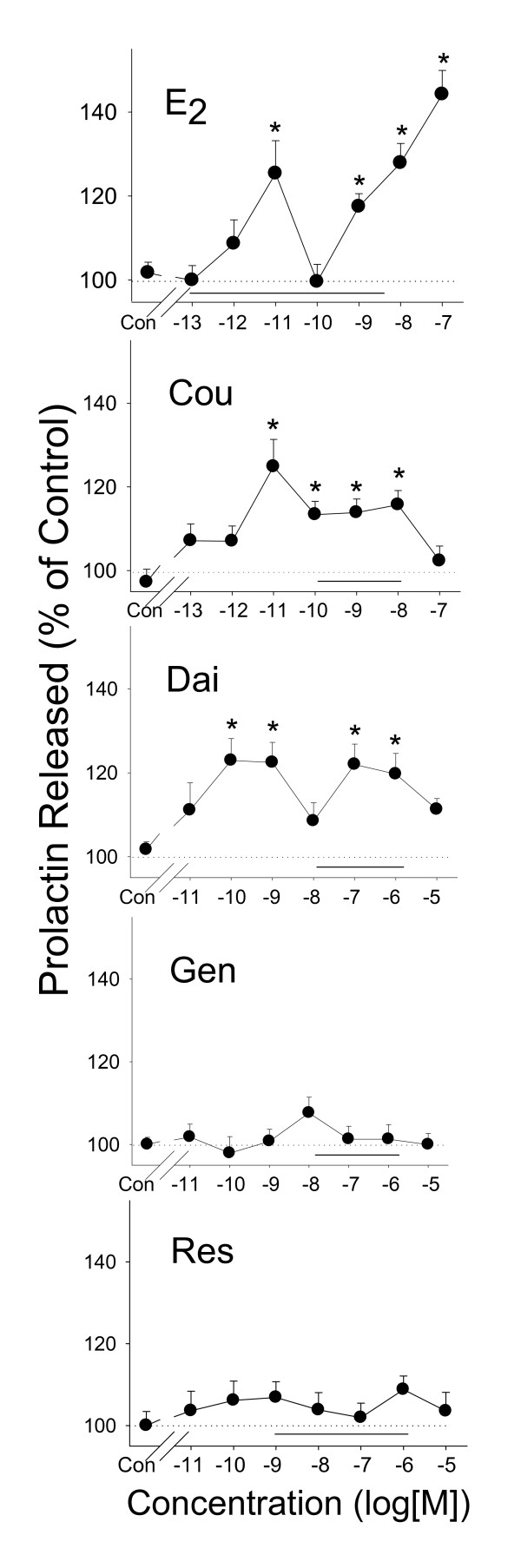
**PRL release induced by phytoestrogens**. Measurements of PRL (by RIA) released into the media after 2 minutes of treatment with different concentrations of estrogens: estradiol (E_2_), coumesterol (Cou), daidzein (Dai), genistein (Gen), and *trans*-resveratrol (Res). The solid horizontal line indicates the range of serum and/or urine concentrations of each phytoestrogen found in humans. * = p < 0.05 compared to vehicle treated-cells (Con).

E_2 _caused a rapid (2 min) PRL release in GH3/B6/F10 cells at concentrations of 10^-11 ^M–10^-7 ^M (except at 10^-10 ^M). Among the phytoestrogens, only coumesterol (at 10^-11^–10^-8 ^M concentrations) and daidezin (at 10^-10^–10^-6 ^M concentrations, except 10^-8 ^M) significantly increased PRL release. Note the interruption of effective doses by an ineffective dose, as we have seen previously [22,36]. Genistein and *trans*-resveratrol did not significantly change PRL release at any concentration tested. In Figure 1 and all following figures showing concentration ranges for responses, the horizontal bars on the graphs depict the range of concentration of these compounds that have been measured in human serum or urine.

### Phosphorylation of ERK1/2 caused by E_2 _and different phytoestrogens (Figure 2)

**Figure 2:**
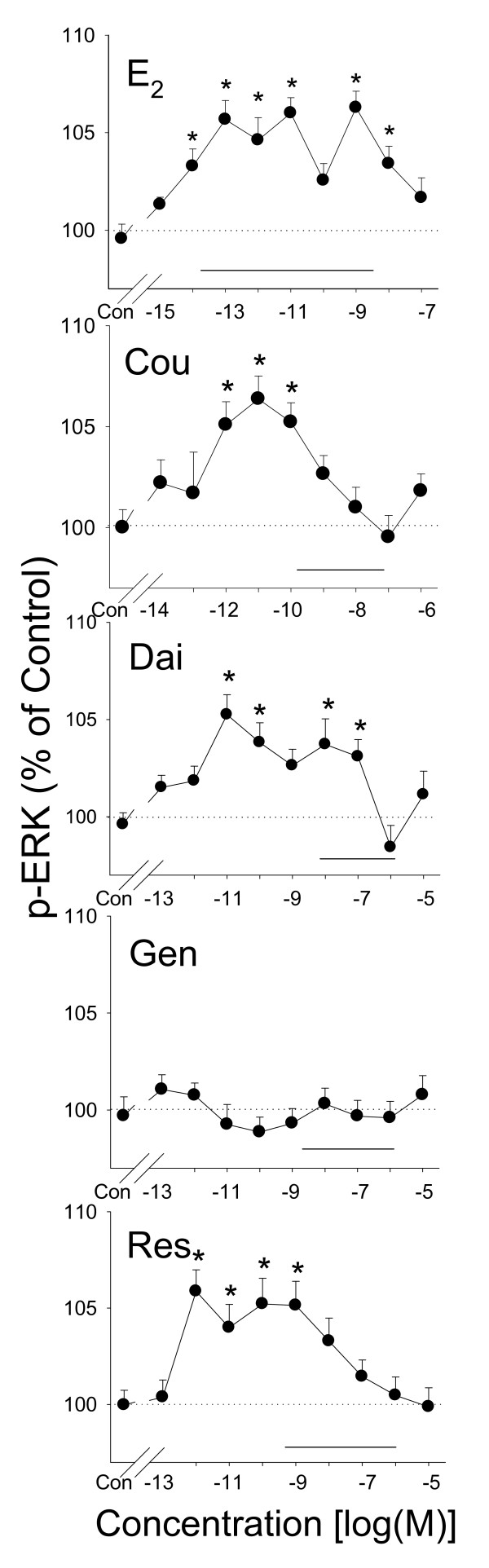
**ERK phosphorylation induced by phytoestrogens**. Quantitative plate assay measurements of phosphorylated ERKs 1 and 2 after 5 minutes of treatment with different concentrations of estrogens: estradiol (E_2_), coumesterol (Cou), daidzein (Dai), genistein (Gen), and *trans*-resveratrol (Res). The solid horizontal line indicates the range of serum and/or urine concentrations of each phytoestrogen found in humans. * = p < 0.05 compared to vehicle treated-cells (Con).

E_2 _and phytoestrogens activated ERK phosphorylation with unique concentration-dependent patterns. At concentrations of 10^-14^–10^-8 ^M (except 10^-10 ^M), E_2 _caused a rapid activation of ERKs within 5 min. The maximum responses occurred at 10^-13 ^and 10^-9 ^M concentrations. Unlike E_2_, coumesterol and *trans*-resveratrol only increased phosphorylation of ERKs at one range of concentrations (10^-12^–10^-10 ^M or 10^-12^–10^-9 ^M, respectively). Daidzein, like E_2_, activated ERKs at 10^-11^–10^-7 ^M, but with slight shifts in effective doses. The closely related soy phytoestrogen genistein did not change phosphorylation of ERKs at any concentration examined.

### Phosphorylation of JNK1/2/3 by E_2 _and different phytoestrogens (Figure 3)

**Figure 3:**
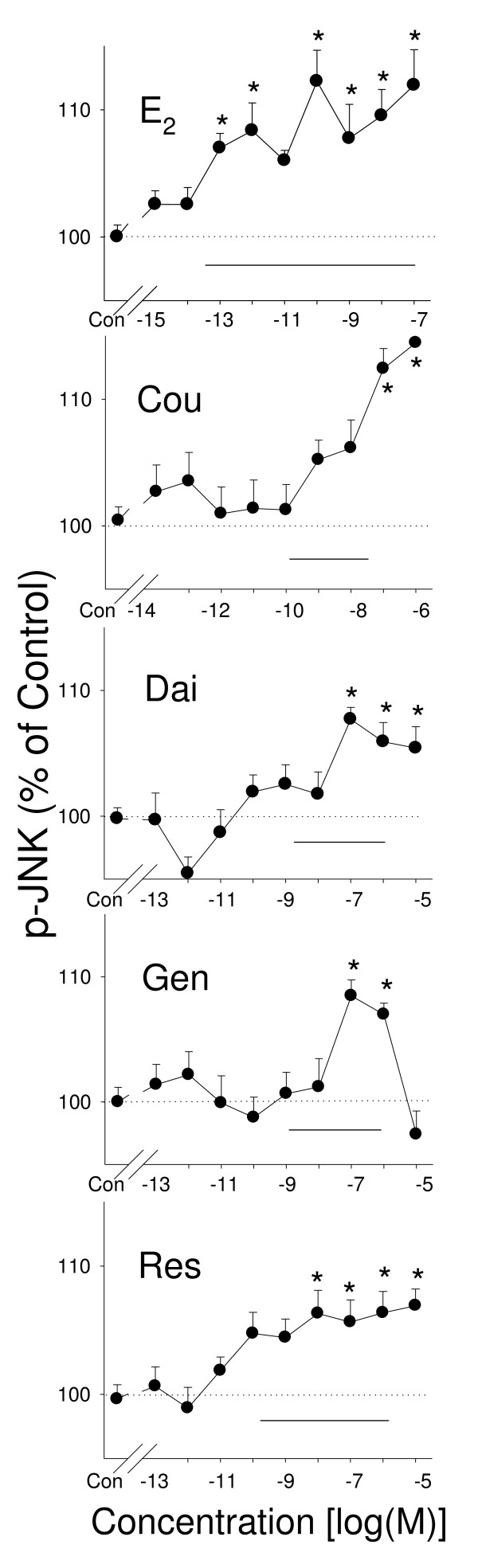
**JNK phosphorylation induced by phytoestrogens**. Quantitative plate assays of phosphorylated JNKs 1, 2 and 3 after 15 minutes of treatment with different concentrations of estrogens: estradiol (E_2_), coumesterol (Cou), daidzein (Dai), genistein (Gen), and *trans*-resveratrol (Res). The solid horizontal line indicates the range of serum and/or urine concentrations of each phytoestrogen found in humans. * = p < 0.05 compared to vehicle treated-cells (Con).

All estrogens increased phosphorylation of a family of JNKs (1/2/3) within 15 minutes, with E_2 _being the most potent at this activity (responding at concentrations as low as 10^-13 ^M). However, again a single intermittent concentration (10^-11 ^M) did not show a significant elevation. Coumesterol, daidzein, genistein, and *trans*-resveratrol increased phosphorylation of JNKs maximally only in the higher concentration ranges (10^-8^–10^-6 ^M).

### E_2 _and different phytoestrogens cause Ca^2+ ^influx

**Figure 4:**
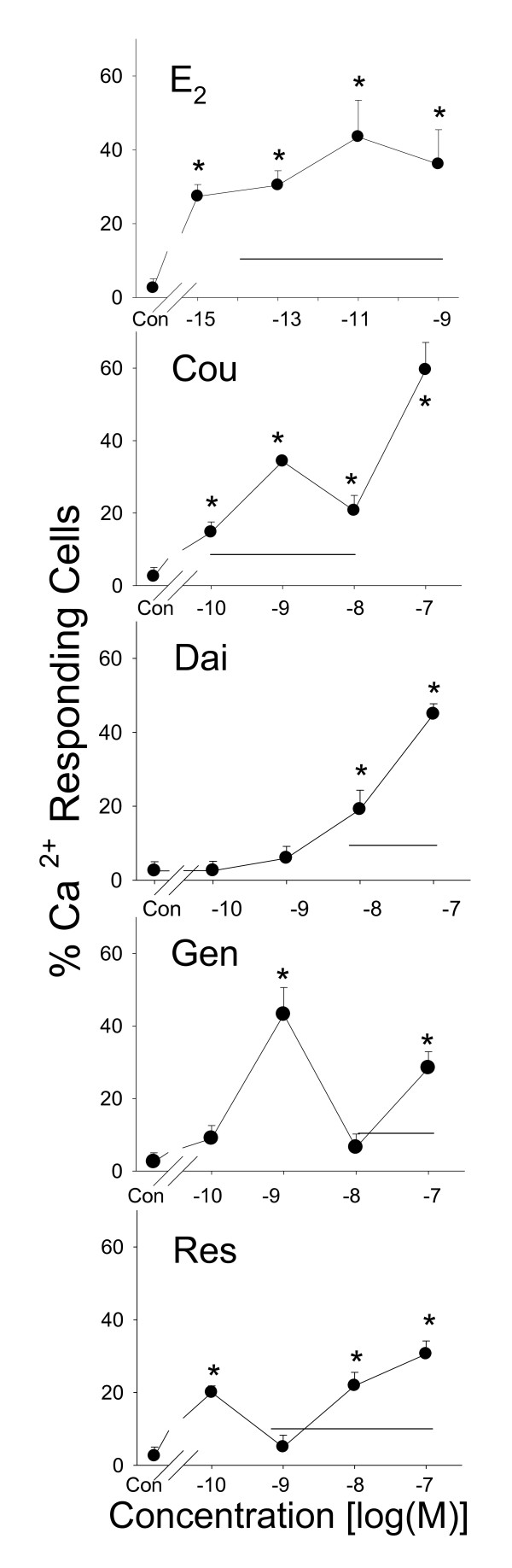
**Percentage of cells showing Ca^2+ ^influx triggered by phytoestrogens**. Fura-2 analyses of the percentage of cells showing a Ca^2+ ^influx response to treatment with different concentrations of estrogens: estradiol (E_2_), coumesterol (Cou), daidzein (Dai), genistein (Gen), and *trans*-resveratrol (Res). The solid horizontal line indicates the range of serum and/or urine concentrations of each phytoestrogen found in humans. * = p < 0.05 compared to vehicle-treated cells (Con).

**Figure 5:**
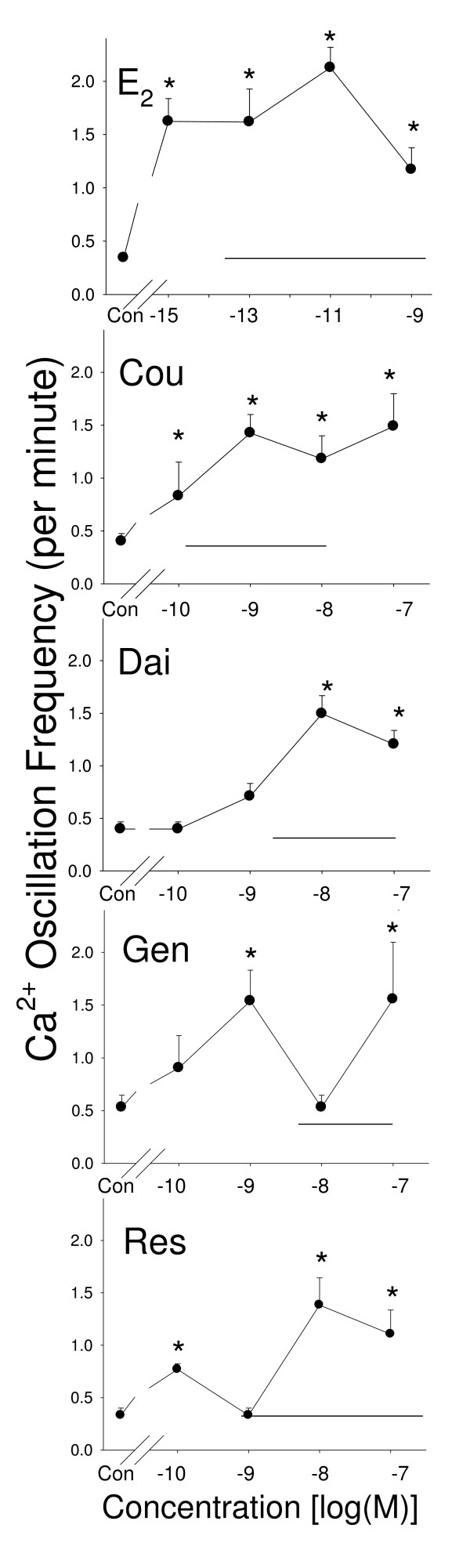
**Effects of phytoestrogens on Ca^2+ ^oscillation**. Fura-2 measurements of Ca^2+ ^oscillation frequency in cells treated with different concentrations of estrogens: estradiol (E_2_), coumesterol (Cou), daidzein (Dai), genistein (Gen), and *trans*-resveratrol (Res). The solid horizontal line indicates the range of serum and/or urine concentrations of each phytoestrogen found in humans. * = p < 0.05 compared to vehicle-treated cells (Con).

**Figure 6:**
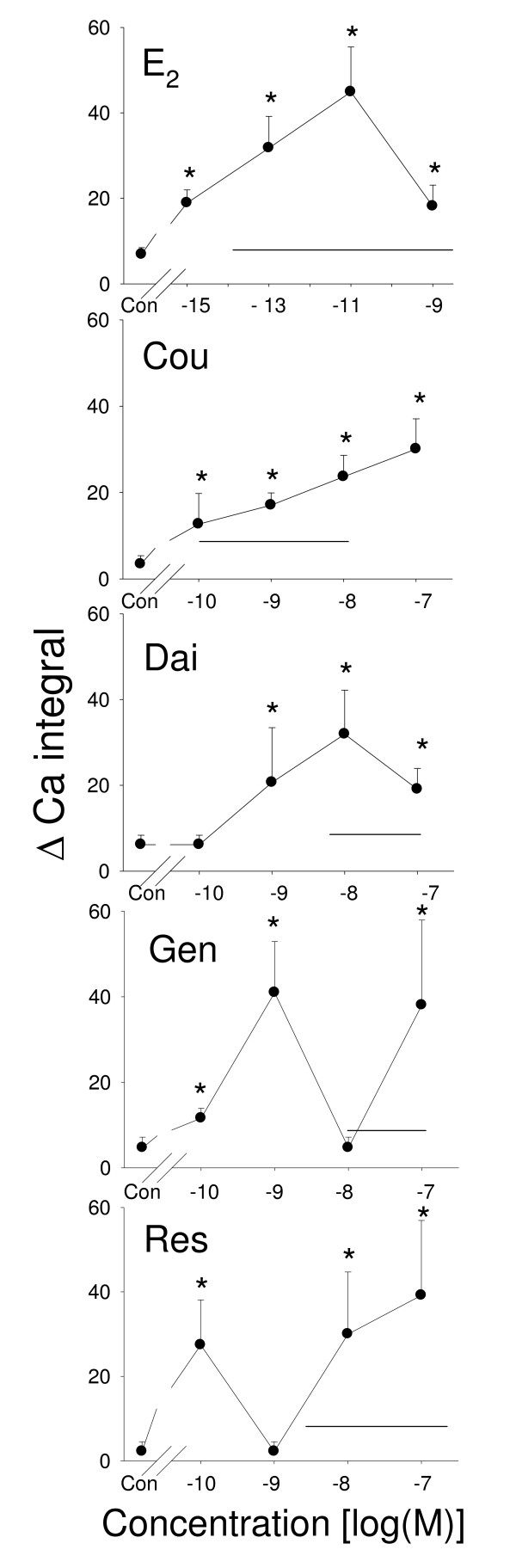
**Effects of phytoestrogens on Ca^2+ ^entry volume**. Fura-2 analyses of the total Ca^2+ ^influx (total area under the Ca^2+ ^curves) in cells treated with different concentrations of estrogens: estradiol (E_2_), coumesterol (Cou), daidzein (Dai), genistein (Gen), and *trans*-resveratrol (Res). The solid horizontal line indicates the range of serum and/or urine concentrations of each phytoestrogen found in humans. * = p < 0.05 compared to vehicle-treated cells (Con).

Approximately 20–40% of the cells showed Ca^2+ ^influx in response to E_2 _treatment regardless of the concentration (10^-15^-10^-9 ^M) used (Figure 4); a similar range of cells responded to coumesterol treatment (10^-10^–10^-7 ^M), but with larger variations between concentrations. Coumesterol caused Ca^2+ ^influx in the highest number of cells (60%) at the highest concentration (10^-7 ^M). For daidzein, only the 10^-8^–10^-7 ^M treatments caused 20–40% of cells to respond. For genistein, only the 10^-9 ^M and 10^-7 ^M concentrations caused Ca^2+ ^influx in 20–40% of cells, but not the intervening 10^-8 ^M concentration.*Trans*-resveratrol at concentrations of 10^-10^–10^-7 ^M caused Ca^2+ ^influx in only ~20% of cells, again with an intervening lack of response at 10^-9 ^M. Treatments with both E_2 _(at 10^–15^-10^-9 ^M) and coumesterol (at 10^-10^–10^-7 ^M) significantly increased the Ca^2+ ^oscillation frequency from 0.4 to 1.5 per min at all concentrations examined (Figure 5). Daidzein, genistein, and *trans*-resveratrol also increased Ca^2+ ^oscillation frequencies at most, but not all concentrations tested. Again, some intermittent concentrations were not effective. The total volume of the Ca^2+ ^influx was measured by adding together the total areas under the in Ca^2+ ^curves for 10 min after treatments (Figure 6). All estrogens caused an increase. For both E_2 _and coumesterol this increase was seen at all concentrations examined. Daidzein, genistein, and *trans*-resveratrol responded at many but not all concentrations, again demonstrating the non-monotonic nature of the responses.

### Rapid nongenomic responses to phytoestrogens and E_2 _are mediated via the mERα (Figure 7)

**Figure 7:**
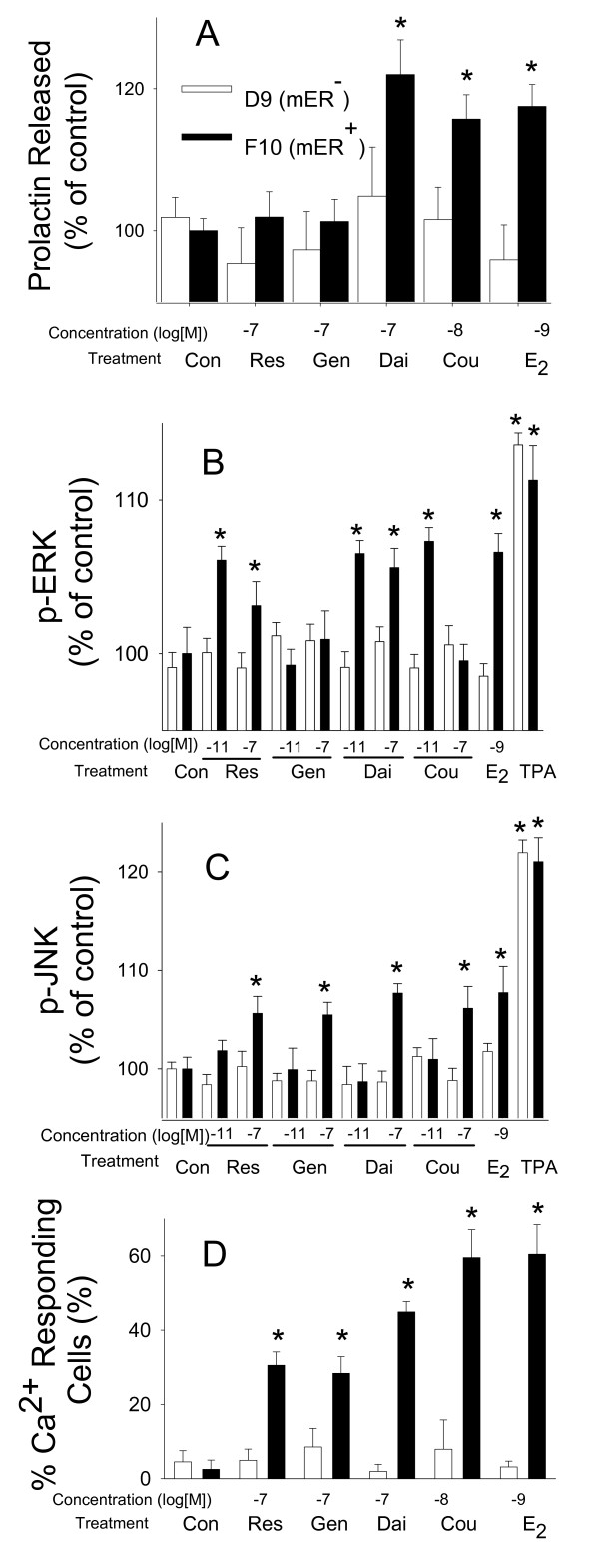
**Involvement of mERα in nongenomic effects by phytoestrogens**. Comparison of mERα-depleted subclone D_9 _(open bars) with mERα-enriched subclone F_10 _cells (solid bars) treated with different estrogens: estradiol (E_2_), coumesterol (Cou), daidzein (Dai), genistein (Gen), and *trans*-resveratrol (Res). (A) PRL released, (B) ERK activation, (C) JNK activation, and (D) % of cells showing Ca^2+ ^influx. * = p < 0.05 compared to vehicle-treated cells (Con).

GH_3 _cell sublines with low (D_9_) or enriched (F_10_) levels of mERα [34,37] showed the expected results for a response dependent upon mERα. The most effective concentrations for each compound for each of the measured responses (PRL release, phosphorylation of ERKs, phosphorylation of JNKs, and percentage of cells showing a Ca^2+ ^response) was used (see Figures 1, 2, 3, 4, 5, 6); each phytoestrogen that had previously evoked a response in F_10 _cells did here as well. However, D_9 _cells could not respond, as expected. Regardless of subclone lineage, the cells are spontaneously active, and respond with a complete Ca^2+ ^response upon KCl depolarization [11]. We also demonstrated that the mERα-dependence for kinase activations affected only the response to estrogens, as 20 nM TPA (phorbol 12-myristate 13-acetate) caused phosphorylation of ERKs and JNKs for both sublines, regardless of mERα status.

## Discussion

Rapid non-genomic effects caused by E_2 _are now well documented in various tissues [9,23,38]; however, those caused by phytoestrogens are little studied. Here we examined the estrogenic activity of four different phytoestrogens commonly available in foods and as food supplements, and demonstrated their abilities to mediate nongenomic actions (rapid PRL release, phosphorylation of ERKs and JNKs, and rapid increases of intracellular Ca^2+ ^levels) in a rat pituitary tumor cell line. We also established a role for mERα in these rapid effects by phytoestrogens by using sublines enriched and depleted for mERα.

Like E_2_, coumesterol and daidzein elicited PRL release at low concentrations, such as those easily attainable in human diets [20,25,27,39]. However, genistein and *trans*-resveratrol were ineffective at all concentrations examined for this response. Although daidzein and genistein are from the same food source and are similar in structure, they bind to ERs with different affinities [40]. Genistein also has effects that are not mediated through ERs, such as inhibition of tyrosine kinases [41], which could alter these signaling pathways through a complex web of signaling interactions. *Trans*-resveratrol can also activate AMP-activated enzymes during caloric restriction [42], and may therefore also initiate other signaling cascades that would impact the signaling machinery that we have focused on here. Thus other responses to these compounds not mediated by ERs could also come into play, depending upon the specific concentrations, and so influence many of the dose-response curves shown here that appear to be nontraditional in shape. It is now becoming commonly accepted that the "web" of signaling outside the focus of individual measurements must be considered when examining these types of responses.

MAPKs control many aspects of mammalian cellular physiology, including cell growth, differentiation and apoptosis. They are well known for responding to mitogens, growth factors (such as epidermal growth factor), and stress [31,43]. We recently demonstrated that other estrogens (both physiological and environmental) can rapidly activate ERKs via mERα and non-genomic pathways in GH_3_/B_6_/F_10 _cells [11,21]. Now several examples from another class of estrogens examined here, phytoestrogens, also increased phosphorylation of ERKs, similar to E_2_. *Trans*-resveratrol had previously been shown to activate ERKs in endothelial cells [44]. Compared to E_2_, which phosphorylated JNKs at sub-picomolar concentrations, these phytoestrogens were less potent, but the activating concentrations required were still well within dietary intake ranges (10^-8^–10^-7 ^M). Activating these MAPKs could lead to changes in both of the opposing effects of cell replication or apoptosis in pituitary cells, depending upon concentrations and combinations, but the precise roles for each of these MAPKs are still unclear [45,46]. It is interesting that the responses of ERKs and JNKs had different sensitivities to this group of estrogens, with JNKs only responding at higher concentrations. If these enzymes were to function differentially in proliferative vs. apoptotic pathways, then such a change in the balance of these competing functions could have profound effects on cell number.

The comparisons between estrogenic compounds' control of intracellular Ca^2+ ^levels were more complex. Most of the phytoestrogens evoked E_2_-like numbers of responding cells, Ca^2+ ^oscillation frequencies, and influx volumes, though E_2 _and coumesterol were more potent. Nevertheless, the effective concentration ranges of phytoestrogens were those found in human serum or urine. Because L-type Ca^2+ ^channel blockers abrogate coumesterol-induced Ca ^2+ ^influx and PRL release [22], other phytoestrogens may do so via the same receptors and mechanisms. Sorting out the exact mechanisms will require using techniques such as patch-clamp recording of Ca^2+ ^currents.

The non-monotonic characteristics of these responses were similar to those we have reported earlier for other estrogens, both physiological and estrogenic environmental contaminants [22,36]. As there are currently disputes in the literature about the effects of phytoestrogens, it is important to study their effects over a wide range of concentrations. These unique dose-response patterns suggest that multiple signaling pathways, receptor couplings, or activations of competing kinases/phosphatases could be involved [6,47,48]. Phytoestrogen exposures and the resulting plasma concentrations vary substantially across human populations and individuals [49] because of genetic variations, differences in dietary intake, and local differences in the phytoestrogen content in plants. The bioavailability of processed phytoestrogens in the gut is also quite different; for example, the total plasma levels of isoflavonoids and lignans in humans range from 10–400 nM, and plasma levels can peak at 2 μM within a few hours of food consumption. There are also reports of individual variations in tissue accumulation of these phytoestrogens such as in prostate [50], and sex variations (female rats show 2.5 times higher concentrations of genistein in liver than males [51] administered the same amounts). These considerations may be important for dosing amounts and schedules if these compounds are to be used effectively as therapeutics.

## Conclusion

Phytoestrogens have rapid and relatively potent non-genomic effects on pituitary cells, apparently mediated via mERα. The concentrations required to activate these effects could easily be attained by dietary levels, and are lower than those required to activate genomic mechanisms. Further knowledge of the multiple factors affecting these responses should include comprehensive examination of the multiple intersecting signaling pathways likely to be evoked. Their usefulness as safe, effective, and inexpensive estrogen replacement therapeutics will have to be carefully considered for each compound individually, as well as how they act in combinations with other dietary, endogenous, and environmental estrogens. Since PRL-secreting adenomas are the most prevalent form of pituitary tumors in human and they contain high levels of ERα mRNA and protein [52], these studies may have implications for the influence of phytoestrogens on the behavior of both normal and tumor tissues.

## Competing interests

The authors declare that they have no competing interests.

## Authors' contributions

YJJ carried out the PRL and kinase experiments in these studies. MYK performed the experiments on intracellular Ca^2+^. All authors participated in the design and analyses of the studies, and wrote, read, and approved the final manuscript.
